# Supplementary figures and images for: Development of a Taqman real-time PCR assay for rapid detection and quantification of Vibrio tapetis in extrapallial fluids of clams
Source: PeerJ. 2015 Dec 22;3:e1484. doi: 10.7717/peerj.1484 (PMC4690387; doi:10.7717/peerj.1484)

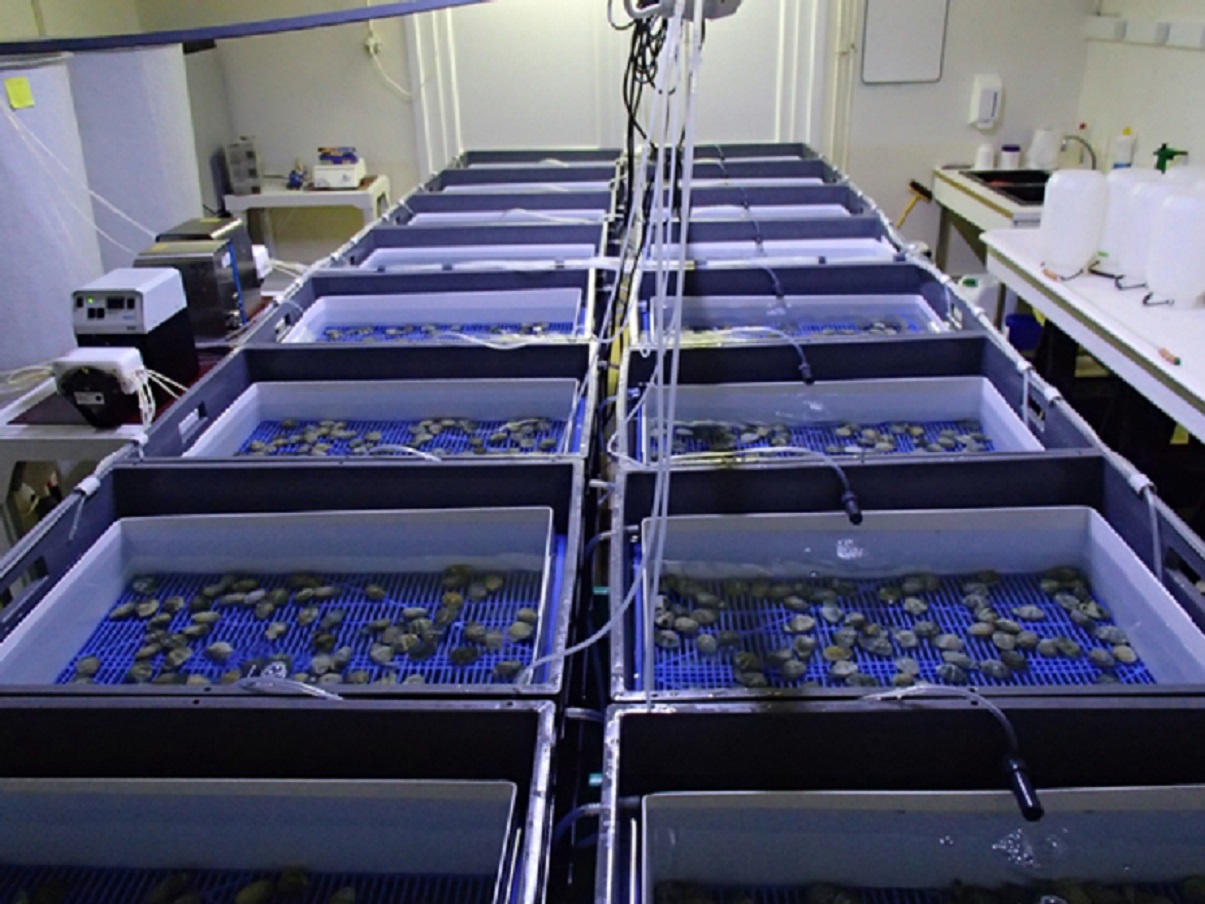

Supplement: Figure S1 [file peerj-03-1484-s001.jpg]
